# Validation and Application of Long-Read Whole-Genome Sequencing for Antimicrobial Resistance Gene Detection and Antimicrobial Susceptibility Testing

**DOI:** 10.1128/aac.01072-22

**Published:** 2022-12-19

**Authors:** Thomas Weinmaier, Rick Conzemius, Yehudit Bergman, Shawna Lewis, Emily B. Jacobs, Pranita D. Tamma, Arne Materna, Johannes Weinberger, Stephan Beisken, Patricia J. Simner

**Affiliations:** a Ares Genetics GmbH, Vienna, Austria; b Department of Pathology, Johns Hopkins University School of Medicine, Baltimore, Maryland, USA; c Department of Pediatrics, Division of Infectious Diseases, Johns Hopkins University School of Medicine, Baltimore, Maryland, USA

**Keywords:** ONT, WGS, WGS-AST, antibiotic resistance, antimicrobial resistance, antimicrobial susceptibility testing, nanopore sequencing, whole-genome sequencing

## Abstract

Next-generation sequencing applications are increasingly used for detection and characterization of antimicrobial-resistant pathogens in clinical settings. Oxford Nanopore Technologies (ONT) sequencing offers advantages for clinical use compared with other sequencing methodologies because it enables real-time basecalling, produces long sequencing reads that increase the ability to correctly assemble DNA fragments, provides short turnaround times, and requires relatively uncomplicated sample preparation. A drawback of ONT sequencing, however, is its lower per-read accuracy than short-read sequencing. We sought to identify best practices in ONT sequencing protocols. As some variability in sequencing results may be introduced by the DNA extraction methodology, we tested three DNA extraction kits across three independent laboratories using a representative set of six bacterial isolates to investigate accuracy and reproducibility of ONT technology. All DNA extraction techniques showed comparable performance; however, the DNeasy PowerSoil Pro kit had the highest sequencing yield. This kit was subsequently applied to 42 sequentially collected bacterial isolates from blood cultures to assess Ares Genetics’s pipelines for predictive whole-genome sequencing antimicrobial susceptibility testing (WGS-AST) performance compared to phenotypic triplicate broth microdilution results. WGS-AST results ranged across the organisms and resulted in an overall categorical agreement of 95% for penicillins, 82.4% for cephalosporins, 76.7% for carbapenems, 86.9% for fluoroquinolones, and 96.2% for aminoglycosides. Very major errors/major errors were 0%/16.7% (penicillins), 11.7%/3.6% (cephalosporins), 0%/24.4% (carbapenems), 2.5%/7.7% (fluoroquinolones), and 0%/4.1% (aminoglycosides), respectively. This work showed that, although additional refinements are necessary, ONT sequencing demonstrates potential as a method to perform WGS-AST on cultured isolates for patient care.

## INTRODUCTION

The rapid pace of antimicrobial resistance (AMR) and its implications as a global threat have been recognized by leading health institutes such as the World Health Organization and the United States Centers for Disease Control and Prevention (1–3). Enhancements to currently available diagnostics are necessary to both limit the spread of drug-resistant pathogens in health care settings and to enable the initiation of early and effective antimicrobial therapy for septic patients (4). Traditional culture-based approaches, even when combined with targeted molecular tests, lead to delays in recognition of AMR pathogens (5). As an alternative, whole-genome sequencing (WGS) technology is increasingly being investigated to optimize patient care.

Advantages of WGS include its ability to both accurately identify pathogens while also providing comprehensive characterization of AMR markers, plasmid replicon types, and virulence factors. The genomic information can also be used for WGS predictive antimicrobial susceptibility testing (WGS-AST). While WGS is being increasingly adopted for epidemiological analysis of bacterial isolates (e.g., surveillance of foodborne pathogens [6, 7]), several limitations hinder its broad-scale adoption. Arguably, the most critical limitations include cost, turnaround time, complexity of testing, the requirement of bioinformatics expertise, and challenges with deploying WGS methodology in low-resource laboratory settings.

The emergence of long-read nanopore sequencing, as marketed by Oxford Nanopore Technologies (ONT), is an alternative to standard short-read sequencing platforms. ONT uses real-time electronic measurements of individual nucleic acid molecules as they pass through protein pores (8). ONT sequencing offers unique advantages over short-read sequencing such as real-time basecalling and analysis, longer sequencing reads (up to several thousand base pairs), lower equipment costs, reductions in turnaround times from days to hours (9–11), feasibility of smaller run sizes, high portability due to the absence of optics and tight integration of detectors with consumables, and less complex sample preparation (12). A disadvantage of ONT sequencing, however, is the high error rate of sequencing reads compared to short-read sequencing. This limitation requires specialized bioinformatics to enhance data quality (13). While the error rate is affected by multiple factors, recent studies indicate that error rates for newer chemistry versions have improved significantly, with ~6% for R9.4.1 and R10.3 (14) compared to ~1% for R10.4 (15).

We present the setup, validation, and application of a workflow for rapid long-read WGS of bacterial isolates and data interpretation via automated bioinformatics integrated in AREScloud. In an initial validation phase, we conducted a three-center study to establish best practice protocols for ONT-based WGS of bacterial isolates, evaluating DNA extraction methods, library preparations, and sequencing steps using approaches offered by different vendors. In a subsequent application phase, the optimal workflow identified in the initial stage was applied to consecutive clinical isolates recovered from blood culture specimens. Finally, we investigated the accuracy of pathogen characterization and WGS-AST applying long-read WGS.

## RESULTS

### Initial validation-phase sequencing and assembly.

The turnaround times for the DNA extraction were approximately 1 h 30 min (PowerSoil), 2 h 20 min (QIAsymphony), and 3 h 30 min (MagAttract), respectively. The batch sizes for the PowerSoil and QIAsymphony extraction methods were 24 isolates, while only 12 isolates were processed in parallel with the MagAttract kit due to limitations with the magnet. The total sequencing yield (total bases) of samples extracted using the PowerSoil protocol was approximately 18% higher than the MagAttract batch and 9% higher than the QIAsymphony batch.

ONT reads had an average base quality Phred score of 11.34 ± 0.58, confirming a high per-base error probability of ONT sequencing (Table S2). The resulting *de novo* genome assemblies consistently exceeded RefSeq minimal assembly quality criteria (16) across all partner labs and extraction methods (Table S3). The average nucleotide identity (ANI) was above 99.5% for all assemblies (Fig. S1) and correlated with sequencing depth (40× to 10×) across the three partner laboratories and three extraction methods (Spearman’s rank-order correlation coefficient, *r* = 0.6, *P* < 0.05 [Fig. S2]). The ANI of the Illumina assemblies compared to the reference genomes was significantly higher than the ANI of the ONT assemblies compared to the reference genomes.

### Initial validation-phase taxonomic identification.

Taxonomic identification resulted in 100% accuracy across extraction methods and laboratories (Table 1). Plasmid replicon detection accuracy was between 87% and 93% for PowerSoil and QIAsymphony. Differences were due to identification of plasmids by the ONT assemblies (e.g., rep13 or IncFII), which were not detected by the corresponding Illumina references. For the MagAttract batch for partner lab B, several additional replicon types were found, resulting in lower specificity. Since this observation was specific to laboratory B, it was presumed to be due to contamination. Plasmid replicon detection based on ONT data performed similar to Illumina data (87%), with accuracies of 87% to 93% (PowerSoil), 55% to 87% (MagAttract), and 87% to 93% (QIAsymphony). The limit of detection (LOD) to achieve an identification was 10× coverage. Typing via core genome multilocus sequence typing (cgMLST) showed high intersample variability with an average distance of 90 ± 50 different loci per scheme. For the six species evaluated, the percentage of discordant loci compared to public ATCC and RefSeq reference genomes was above the acceptable cgMLST schema-specific complex type distance. Higher distances are generally caused by the higher error rate of ONT sequencing than Illumina sequencing, which leads to misassembled loci in resulting assemblies (17). Reproducibility across labs and extraction kits was consistently high for pathogen detection (100%) and plasmid replicon detection (100%).

**TABLE 1:** Accuracy and reproducibility of the assays performed on the validation samples

Partner lab	Extraction method	Identification (% [no. of samples/total no. of samples])	cgMLST result (% [no. of samples/total no. of samples])	AMR marker detection (% [no. of markers/total no. of markers])	WGS-AST (range [%])	Plasmid replicon detection (% [no. of replicons/total no. of replicons])
AG^*a*^	PowerSoil	100 (6/6)	66 (4/6)	97 (362/373)	97 ± 5.6	87 (13/15)
A	PowerSoil	100 (6/6)	0 (0/6)	96 (358/373)	97 ± 5.6	93 (14/15)
B	PowerSoil	100 (6/6)	0 (0/6)	94 (350/373)	95 ± 5.9	87 (13/15)
C	PowerSoil	100 (6/6)	0 (0/6)	93 (348/373)	90 ± 11.8	87 (13/15)
A	MagAttract	100 (6/6)	0 (0/6)	93 (347/373)	97 ± 5.6	87 (13/15)
B	MagAttract	100 (6/6)	0 (0/6)	94 (351/373)	95 ± 5.9	55 (8/15)
C	MagAttract	100 (6/6)	0 (0/6)	92 (343/373)	96 ± 6.4	80 (12/15)
A	QIAsymphony	100 (6/6)	0 (0/6)	94 (351/373)	97 ± 5.6	93 (14/15)
B	QIAsymphony	100 (6/6)	0 (0/6)	94 (350/373)	97 ± 5.6	87 (13/15)
C	QIAsymphony	100 (6/6)	0 (0/6)	95 (354/373)	97 ± 5.6	87 (13/15)
Reproducibility		100 (54/54)	0 (0/54)	97 (2,489/2,556)	96 (175/182)	100 (72/72)

a AG, Ares Genetics.

### Initial validation-phase AMR marker detection.

The accuracy for AMR marker detection based on ONT data was slightly lower than Illumina data (97%) and ranged between 93% and 96% (PowerSoil), 92% to 94% (MagAttract), and 94% to 95% (QIAsymphony) for samples using the three different DNA extraction methods. The lower accuracy of ONT AMR marker detection was due to fewer true-positive AMR markers detected for ONT compared to Illumina, leading to a lower sensitivity. The reproducibility of AMR marker detection within ONT data was high across all samples (97%). Any differences were in part due to allele variants, e.g., *tet38* in Staphylococcus aureus or *adeB* in Acinetobacter baumannii, for which two allele variants were detected across the three partner labs.

### Initial validation-phase predictive WGS-AST.

Performance metrics for WGS-AST based on ONT data were not significantly different from Illumina data (see Table S4 in the supplemental material). Categorical agreement (CA) was as follows across extraction methods and partner labs: A. baumannii, 100%; Enterococcus faecium, 100%; Klebsiella quasipneumoniae, 60%; S. aureus, 88%; E. coli, 96%; and Pseudomonas aeruginosa, 77%. For P. aeruginosa (ID245), resistance was overestimated for the MagAttract batch across vendors, resulting in a CA ranging from 25% to 100%. Resistance overestimation in ONT samples compared to Illumina data was observed for P. aeruginosa (ID245) (25% to 75% major error [ME]) and E. coli (ID248) (8.3% to 25% ME). At 30× ONT coverage, WGS-AST results stabilized across vendors and extraction methods.

### Subsequent application-phase sequencing and assembly.

Sequencing run times to reach 30× coverage ranged from 3.5 h to 23.5 h (6.5 to 9.5 h for A. baumannii, 6 to 12 h for E. coli, 6.5 to 15 h for E. faecium, 4.5 to 7.5 h for S. aureus, 3.5 to 6.5 h for K. pneumoniae, and 8 to 23.5 h for P. aeruginosa [data not shown]). Basecalling quality of the ONT application data set was comparable to the validation set (average Phred score, 11.47 for the application set compared to 11.43 for the validation set) but still showed a higher per-base error probability of ONT sequencing than Illumina (Table S5). The quality metrics of the resulting *de novo* genome assemblies were consistently high for all samples (Table S6).

### Subsequent application-phase taxonomic identification.

All isolates were assigned the correct species as determined by matrix-assisted laser desorption ionization–time of flight mass spectrometry (MALDI-TOF MS).

### Subsequent application-phase AMR markers.

Extended-spectrum beta-lactamase (ESBL) genes were identified in three E. coli isolates (*bla*_CTX-M-15_, *bla*_CTX-M-27_), three K. pneumoniae isolates (*bla*_CTX-M-15_), and a P. aeruginosa isolate (*bla*_GES-7_) (Table S7). One A. baumannii isolate (Abau_bc09) harbored the *bla*_OXA-23_ carbapenemase gene, while both harbored *bla*_OXA-51_-like genes (*bla*_OXA-66_, *bla*_OXA-180_). Abau_bc09 also contained an upstream ISAba1 gene, suggesting high expression and carbapenem resistance. An *ampC* gene was identified in one A. baumannii isolate (*bla*_ADC-158_). Eight distinct Pseudomonas-derived cephalosporinase (PDC) genes were found in the P. aeruginosa isolates. Additional AMR markers identified in Gram-negative organisms are described in Table S7. Among the S. aureus isolates, *mecA* genes were identified in 4 of 5 isolates. Genes of the *vanA* cluster were detected in 2 of 5 E. faecium isolates.

### Subsequent application-phase antimicrobial susceptibility testing.

Genomic AST results were generated for 64 species-antimicrobial pairs (Table S8). A WGS-AST evaluation of the 444 species-antimicrobial pairs is displayed in Table 2. The CA per species ranged between 81.7% (K. pneumoniae) and 100% (S. aureus), very major error (VME) ranged from 0% (E. coli, P. aeruginosa, and S. aureus) to 18.8% (A. baumannii), and major error (ME) ranged from 0% (A. baumannii and S. aureus) to 17.2% (K. pneumoniae). Overall, CA was 95% for penicillins, 82.4% for cephalosporins, 76.7% for carbapenems, 86.9% for fluoroquinolones, and 96% for aminoglycosides. VME and ME were 0% and 16.7% (penicillins), 11.7% and 3.6% (cephalosporins), 0% and 24.4% (carbapenems), 2.5% and 7.7% (fluoroquinolones), and 0% and 4.1% (aminoglycosides).

**TABLE 2:** WGS-AST results compared to phenotypic BMD-AST results grouped by species for application-phase samples^*a*^

Organism	CA (%)	VME (%)	ME (%)	mE (%)	TN^*b*^	FP^*b*^	FN^*b*^	TP^*b*^	I→S^*b*^	I→R^*b*^	Total^*b*^
All (*n* = 42)	87.6	4.8	10.6	3.8	269	32	6	120	3	14	444
Acinetobacter baumannii (*n* = 2)	87.0	18.8	0.0	0.0	7	0	3	13	0	0	23
Enterococcus faecium (*n* = 5)	81.8	15.4	5.9	9.1	16	1	2	11	1	2	33
Escherichia coli (*n* = 10)	87.2	0.0	12.6	3.8	97	14	0	39	0	6	156
Klebsiella pneumoniae (*n* = 10)	81.7	3.7	17.2	4.8	77	16	1	26	0	6	126
Pseudomonas aeruginosa (*n* = 10)	94.6	0.0	2.4	3.6	41	1	0	12	2	0	56
Staphylococcus aureus (*n* = 5)	100.0	0.0	0.0	0.0	31	0	0	19	0	0	50

a CA, categorical agreement; VME, very major error; ME, major error; mE, minor error; TN, true negative; FP, false positive; FN, false negative; TP, true positive; I→S, intermediate phenotype predicted as susceptible; I→R, intermediate phenotype predicted as resistant.

b Data represent number of samples.

Evaluating VMEs by species (Tables 3 and 4), A. baumannii isolate Eclo-Abau_bc10 had VMEs for piperacillin-tazobactam, ceftazidime, and cefepime, while the other A. baumannii isolate (Eclo-Abau_bc09) had no VMEs. In the 10 E. coli isolates, 3 isolates had 100% WGS-AST agreement. In 4 isolates, carbapenem resistance was overestimated, resulting in MEs, and in 2 isolates, piperacillin-tazobactam resistance was overestimated. Two E. coli isolates were overestimated for amikacin, and one isolate was overestimated for ciprofloxacin, gentamicin, and tetracycline. Three out of 5 E. faecium strains had 100% agreement; one strain had an ME for ampicillin resistance and a VME for ciprofloxacin resistance; another strain had a VME for linezolid resistance. All WGS-AST predictions for S. aureus isolates were in agreement. Among 10 K. pneumoniae isolates, 3 MEs for carbapenems were identified. Among the 10 K. pneumoniae isolates, overestimation of resistance resulted in 5 MEs for tetracycline, 2 MEs for ciprofloxacin, and 1 ME for ceftazidime. Resistance underestimation for cefazolin resulted in a VME in 1 K. pneumoniae isolate. For P. aeruginosa isolates, resistance to gentamicin was overestimated in 1 isolate. The three E. coli isolates with several AMR markers (Ecol_bc01, Ecol_bc09, and Ecol_bc10) showed the highest number of MEs among E. coli isolates, generally to ertapenem and the aminoglycosides.

**TABLE 3:** Comparison of WGS-AST results to BMD-AST results and detected AMR markers for β-lactamases in Gram-negative isolates of the application phase

Isolate ID	Pathogen	β-Lactamase AMR markers	Resistance to^*a*^:																
			AMP	AMP-SLB	PTZ	ERT	MER	AZT	CFZ	CAZ	CRO	FEP	LEV	CIP	TOB	GEN	AMI	TET	TSU
Eclo-Abau_bc09	A. baumannii	OXA-66, OXA-23		R/R	R/R					R/R	R/R	R/R	R/R	R/R	R/R	R/R	R/R	R/R	R/R
Eclo-Abau_bc10	A. baumannii	ADC-158, OXA-180		S/−	S/R					S/R	R/R	S/R	S/S	S/S	S/S	S/S	S/S	S/S	S/S
Ecol_bc01	E. coli	AmpH, CTX-M-15, EC-145, OXA-1, TEM-1	R/R	R/I	**R/S**	**R/S**		R/R	R/R	R/I	R/R	R/−	R/R	R/R	R/R	R/R	**R/S**	R/R	R/R
Ecol_bc02	E. coli	EC-145, AmpC1	S/S	S/S	S/S	S/S		S/S	S/S	S/S	S/S	S/S	S/S	S/S	S/S	S/S	S/S	S/S	S/S
Ecol_bc03	E. coli	AmpH, EC-145, TEM-1	R/R	R/I	S/S	**R/S**		S/S	R/−	S/S	S/S	S/S	S/S	S/S	S/S	S/S	S/S	S/S	S/S
Ecol_bc04	E. coli	AmpH, EC-145, TEM-1	R/R	R/R	**R/S**	S/S		S/S	R/−	S/S	S/S	S/S	S/S	S/S	S/S	S/S	S/S	S/S	S/S
Ecol_bc05	E. coli	AmpC1, AmpH, EC-145, TEM-84, TEM-1	R/R	**R/S**	S/S	S/S		S/S	S/S	S/S	S/S	S/S	S/S	S/S	S/S	S/S	S/S	S/S	S/S
Ecol_bc06	E. coli	AmpH, TEM-1	R/R	R/R	S/S	S/S		S/S	R/R	**R/S**	S/S	S/S	S/S	R/I	S/S	S/S	S/S	R/R	R/R
Ecol_bc07	E. coli	AmpH, EC-145, TEM-1	R/R	R/I	S/S	S/S		S/S	R/I	S/S	S/S	S/S	S/S	S/S	S/S	S/S	S/S	S/S	S/S
Ecol_bc08	E. coli	AmpH, EC-145, AmpC1	S/S	S/S	S/S	S/S		S/S	S/S	S/S	S/S	S/S	S/S	S/S	S/S	S/S	S/S	S/S	S/S
Ecol_bc09	E. coli	AmpH, EC-145, OXA-1, CTX-M-15	R/R	R/−	**R/S**	**R/S**		R/R	R/R	R/R	R/R	R/R	R/R	R/R	R/R	R/R	**R/S**	R/R	S/S
Ecol_bc10	E. coli	AmpH, EC-145, CTX-M-27	R/R	R/R	S/S	**R/S**		R/R	R/R	R/R	R/R	R/R	R/R	**R/S**	S/S	**R/S**	S/S	**R/S**	S/S
Kpne_bc01	K. pneumoniae	SHV-11				S/S		S/S	S/S	S/S	S/S	S/S	S/S	S/S	S/S	S/S	S/S	R/R	**R/S**
Kpne_bc02	K. pneumoniae	SHV-112, LEN-6				S/S		S/S	S/S	S/S	S/S	S/S	S/S	S/S	S/S	S/S	S/S	**R/S**	S/S
Kpne_bc03	K. pneumoniae	CTX-M-15, SHV-1, TEM-1				**R/S**		R/R	R/R	R/R	R/R	R/I	**R/S**	R/I	S/S	S/S	S/S	R/R	R/R
Kpne_bc04	K. pneumoniae	SHV-26				S/S		S/S	S/S	S/S	S/S	S/S	S/S	**R/S**	S/S	S/S	S/S	**R/S**	S/S
Kpne_bc05	K. pneumoniae	KPC-2, SHV-27, TEM-1				R/R		R/R	R/R	**R/S**	R/R	S/S	**R/S**	**R/S**	S/S	S/S	S/S	**R/S**	S/S
Kpne_bc06	K. pneumoniae	SHV-28, SHV-1				S/S		S/S	R/R	S/S	S/S	S/S	S/S	S/S	S/S	S/S	S/S	**R/S**	S/S
Kpne_bc07	K. pneumoniae	SHV-11, CTX-M-15				**R/S**		R/R	R/R	R/-	R/R	R/I	**R/S**	R/-	S/S	S/S	S/S	R/R	R/R
Kpne_bc08	K. pneumoniae	CTX-M-15, SHV-11, TEM-1				**R/S**		R/R	R/R	R/R	R/R	R/I	R/I	R/R	**R/S**	R/R	S/S	**R/S**	R/R
Kpne_bc09	K. pneumoniae	SHV-1				S/S		S/S	R/I	S/S	S/S	S/S	S/S	S/S	S/S	S/S	S/S	R/R	S/S
Kpne_bc10	K. pneumoniae	SHV-11				S/S		S/S	S/R	S/S	S/S	S/S	S/−	S/−	S/S	S/S	S/S	R/R	S/S
Paer_bc01	P. aeruginosa	OXA-494, PDC-1					S/S						S/I	S/S	S/S	S/S	S/S		
Paer_bc02	P. aeruginosa	OXA-488, PDC-38					S/S						S/S	S/S	S/S	S/S	S/S		
Paer_bc03	P. aeruginosa	OXA-494, PDC-8					S/−						S/I	S/S	S/S	S/S	S/S		
Paer_bc04	P. aeruginosa	OXA-395, PDC-3					R/−						S/S	S/S	S/S	**R/S**	S/S		
Paer_bc05	P. aeruginosa	OXA-488, PDC-275, GES-7					R/R						R/R	R/R	R/R	R/R	R/R		
Paer_bc06	P. aeruginosa	OXA-486, PDC-257					S/S						S/S	S/S	S/S	S/S	S/S		
Paer_bc07	P. aeruginosa	OXA-488, PDC-5					R/R						R/R	R/R	S/S	S/S	S/S		
Paer_bc08	P. aeruginosa	OXA-494, PDC-8					S/S						S/S	S/S	S/S	S/S	S/S		
Paer_bc09	P. aeruginosa	PDC-3, OXA-50					R/R						R/R	R/R	S/S	S/S	S/S		
Paer_bc10	P. aeruginosa	OXA-494, PDC-31					S/−						S/−	S/S	S/S	S/S	S/S		

a WGS-AST results/BMD-AST results; very major errors (S/R) are colored in dark gray, major errors (R/S) in bold, and minor errors (R/I and S/I) underlined. −, missing AST result due to discordant interpretations of the triplicate BMD-AST measurements.

**TABLE 4:** Comparison of WGS-AST results to BMD-AST results and detected AMR markers for β-lactamases in Gram-positive isolates of the application phase

Isolate ID	Pathogen	β-Lactamase AMR markers	Resistance to^*a*^:										
			AMP	CIP	ERY	GEN	LIN	SYN	TET	VAN	PEN	LEV	OXA
Efae-Saur_bc01	E. faecium		R/R	R/R	R/R		S/R	S/S	S/S	R/R			
Efae-Saur_bc02	E. faecium		S/S	S/S	R/−		S/−	S/I	S/S	S/S			
Efae-Saur_bc03	E. faecium		**R/S**	S/R	R/I		S/S	S/S	S/S	S/S			
Efae-Saur_bc04	E. faecium		R/R	R/R	R/I		S/S	S/S	R/R	S/S			
Efae-Saur_bc05	E. faecium		R/R	R/R	R/R		S/S	S/S	S/S	R/R			
Efae-Saur_bc06	S. aureus	*blaZ*		R/R	R/R	S/S	S/S	S/S	S/S	S/S	R/R	R/R	R/R
Efae-Saur_bc07	S. aureus			R/R	S/S	S/S	S/S	S/S	S/S	S/S	R/R	R/R	R/R
Efae-Saur_bc08	S. aureus	*blaZ*		S/S	R/R	S/S	S/S	S/S	S/S	S/S	R/R	S/S	R/R
Efae-Saur_bc09	S. aureus	*blaZ*		R/R	R/R	S/S	S/S	S/S	S/S	S/S	R/R	R/R	R/R
Efae-Saur_bc10	S. aureus	*blaZ*		S/S	R/R	S/S	S/S	S/S	S/S	S/S	R/R	S/S	S/S

a WGS-AST results/BMD-AST results; very major errors (S/R) are colored in gray, major errors (R/S) in bold, and minor errors (R/I and S/I) underlined. −, missing AST result due to discordant interpretations of the triplicate BMD-AST measurements.

## DISCUSSION

We sought to identify best practices in sequencing protocols for ONT sequencing for predictive WGS-AST. During an initial validation phase, we found no significant differences in assay performance for three different DNA extraction methods used by each of three partner laboratories. However, due to the higher sequencing yield (PowerSoil yield was 18% higher than MagAttract and 9% higher than QIAsymphony) and faster turnaround time (PowerSoil, 1 h 30 min; QIAsymphony, 2 h 20 min; MagAttract, 3 h 30 min), we elected to use the PowerSoil extraction method for a subsequent application phase applying methodologies from the first phase to the testing of 42 blood culture isolates. Overall, and in agreement with others (18, 19), we found that ONT data performed comparably to Illumina data except for cgMLST, in which the higher error rates of ONT sequencing resulted in inflated phylogenetic distances between samples. The validation phase showed that 30× coverage is sufficient for bioinformatics data analysis; this coverage was achieved after 3.5 to 23.5 h for the application-phase samples. Average sequencing run times to achieve 30× coverage varied between species, with K. pneumoniae showing among the shortest run times, between 3.5 and 6.5 h. These times are comparable to the times reported in a previous study (20) and result in a similar total time from sample to AMR marker detection as the Nanopore assembly approach described in that study. While this observation needs to be confirmed in further studies, it indicates that sequencing run times for clinical diagnostics can be significantly shortened from the standard 72 h without losing resolution.

An interesting finding from our study was that one of the extraction kits evaluated demonstrated higher levels of predicted resistance by WGS-AST with the P. aeruginosa isolate across partner laboratories, especially when there was low sequence coverage. This points to the potential of nucleic acid contaminants from the kit and reagents impacting WGS-AST results. However, no contamination with Pseudomonas was observed in the no-template extraction control. The impact on sequencing reagent contaminants is well described for identification and microbiome assessment (21–23). Pseudomonas species are known to be a common kit and reagent contaminant, and it is plausible that contaminating nucleic acid from these organisms impacts WGS-AST predictions. Consistent with this observation is that with increasing sequencing coverage (≥30× coverage) of the isolate of interest, the discrepancies are no longer evident. Thus, quality controls for sequencing coverage are important to assess completeness of the resistome but also to ensure that contaminating nucleic acid from kits does lead to discrepant WGS-AST results.

During the validation phase, ONT assemblies performed similarly to Illumina for predictive WGS-AST. However, false-positive resistance WGS-AST results (MEs) occurred with ONT sequencing with greater frequency than Illumina. These false-positive resistant results (3.4% ME over all samples) can be explained by sequencing or assembly issues, e.g., due to contamination or basecalling errors, that occurred across all partner labs and extraction methods. As an example, E. coli isolate ID248 showed low CA due to MEs for amoxicillin-clavulanic acid, cefepime, cefotaxime, and trimethoprim-sulfamethoxazole. These MEs were likely related to genomic contigs from possible library contaminations: assembly QC and plasmid replicon detection results indicated a contamination with an E. coli strain that differs from the reference species (data not shown).

In line with previous reports, species identification between MALDI-TOF MS and WGS was concordant in the workflow application phase for the organisms included in this study (24). WGS provided higher resolution of identifications (e.g., A. baumannii complex by MALDI-TOF MS were identified as A. baumannii sensu stricto by WGS). WGS-AST performed well for most pathogen compound pairs, with a few notable exceptions, including MEs for tetracycline and levofloxacin in K. pneumoniae (20) (5 and 3 MEs out of 10 isolates, respectively) and ertapenem in E. coli (4 MEs out of 10 isolates). P. aeruginosa and S. aureus performed best, with 0% VME and overall CAs of 94.6% and 100%, respectively. In terms of antimicrobial classes and subclasses, the penicillins, monobactams, glycopeptides, and aminoglycosides all showed a VME of 0% and CA above 95%. While the overall performance of cephalosporins was mixed due to issues with one A. baumannii isolate, the third-generation cephalosporin, ceftriaxone, showed a CA of 100% and VME of 0%. Also, known correlations among aminoglycosides and fluoroquinolones were confirmed by the current results, i.e., levofloxacin resistance is positively correlated with ciprofloxacin. While overall categorical agreement was 87.6%, if all intermediate results from broth microdilution antimicrobial susceptibility testing (BMD-AST) were treated as resistant, the observed minor error (mE) would drop to 0%, and CA would improve to 90.8%, with a VME of 6.3%.

Overall, WGS-AST predictions tended to overcall resistance, leading to ME. While error rates need to be lowered to be within acceptable ranges, the results show the potential of WGS-AST to be applied in a clinical setting. A potential approach to mitigate these errors is to introduce an indeterminate or gray zone category for predictions that are not clearly susceptible or resistant based on the prediction pipeline, analogous to the area of technical uncertainty introduced by EUCAST for phenotypic AST results. In addition, recent releases of the ONT chemistry, e.g., R10.4/Kit12, have been shown to have an improved basecalling fidelity (15). This will likely improve assembly quality and reduce overall error rates as genetic AMR features are resolved more accurately. WGS-AST was applied to sequentially collected isolates of the six species of interest without specific sample collection inclusion criteria. This is one of the first studies to validate and assess WGS-AST accuracy based only on long-read sequencing data for clinical isolates from multiple species. Limitations of this study include a small number of isolates to assess WGS-AST accuracy. Further studies using a larger cohort are required.

We established an ONT sequencing workflow and validated its performance for the characterization of bacterial pathogens, specifically for WGS-AST prediction. We demonstrated that a validated ONT sequencing workflow can be systematically and successfully used for WGS-AST on a set of sequentially collected clinical samples recovered from positive blood cultures. However, ongoing optimizations of the ONT sequencing platform are needed to enhance sequencing fidelity, which, in turn, may enable higher predictive accuracy. These developments and the fast turnaround time make ONT a promising platform for AMR detection and WGS-AST for clinical use.

## MATERIALS AND METHODS

### Initial validation phase.

**FIG 1:**
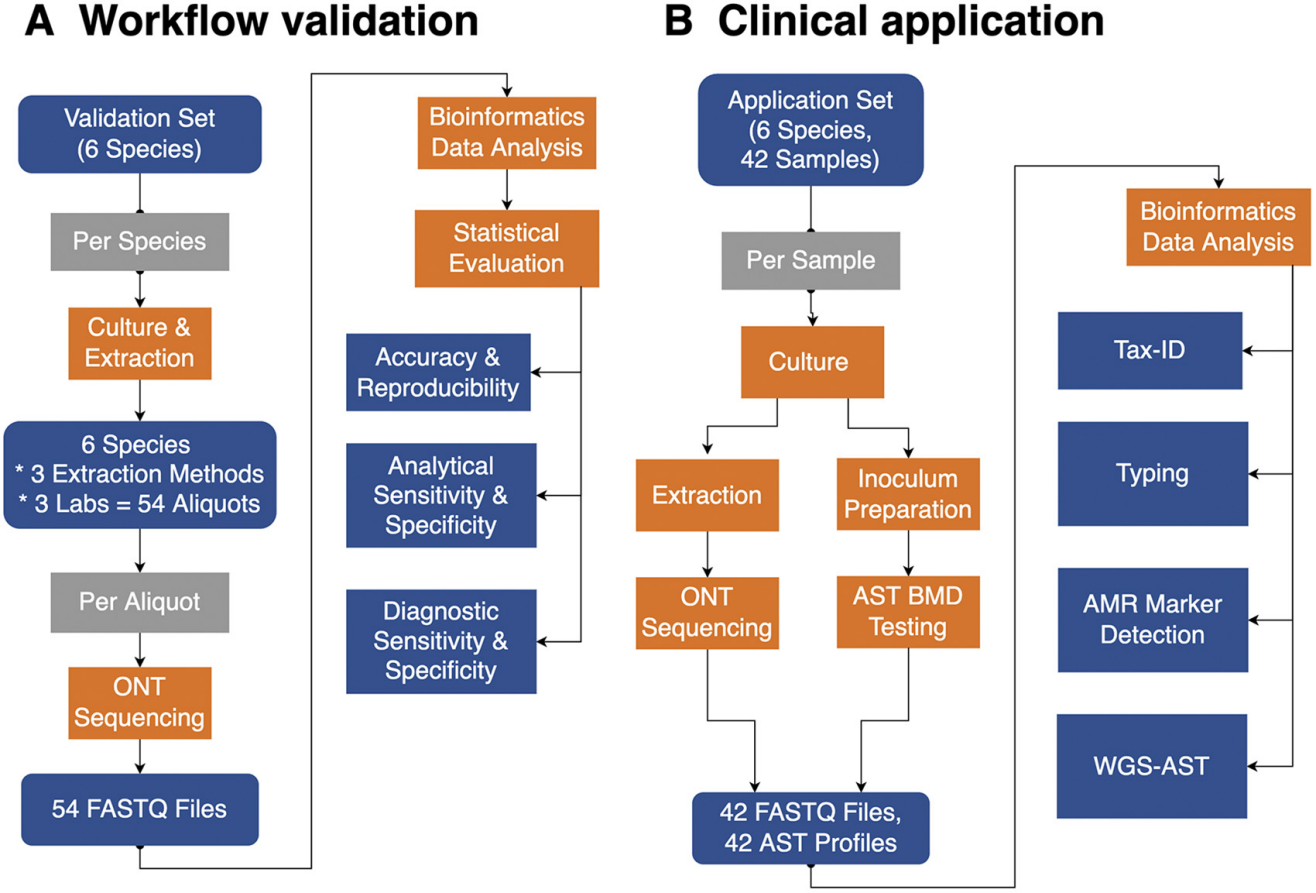
Study workflow for the workflow validation phase (A) and clinical application phase (B).

Three DNA extraction kits were tested across three independent laboratories using a representative set of six bacterial isolates to investigate the accuracy and reproducibility of ONT technology (Fig. 1). Isolate cultures were purchased from Microbiologics (MN, USA). Published reference genomes for each species were used as comparators for assay validation and retrieved either from NCBI RefSeq or the ATCC Genome Portal (available at https://genomes.atcc.org/). Previously acquired Illumina short-read data were used as an additional reference (25).

### Initial validation-phase bacterial culture and DNA extraction.

The six bacterial reference isolates included in the initial validation phase consisted of the following: Acinetobacter baumannii, Enterococcus faecium, Klebsiella quasipneumoniae, Staphylococcus aureus, Pseudomonas aeruginosa, and Escherichia coli (see Table S1 in the supplemental material). Bacterial isolates from frozen stock were serially subcultured twice on casein-peptone soymeal-peptone (CASO) agar (Carl Roth, Karlsruhe, Germany) for 18 h at 37°C. DNA from a 10-μL loop of bacterial culture was extracted using three different methods each, two manual and one automated method.

Two manual extractions were performed using the DNeasy PowerSoil Pro kit (Qiagen, Hilden, Germany) and the MagAttract high-molecular-weight (HMW) DNA kit (Qiagen) with the following modifications: with the PowerSoil method, the isolate was resuspended in buffer CD1, bead beating was done on a PowerLyzer 24 homogenizer (Qiagen), and DNA was eluted in nuclease-free water, while with the HMW method, cultures were resuspended in buffer ATL, and DNA was eluted in water.

An automated extraction was performed using the QIAsymphony SP system (Qiagen) using the QIAsymphony DSP DNA minikit (Qiagen) with the following modifications: cultures were resuspended in 480 μL of buffer P1; off-board lysis was carried out using 21 μL of a 100-mg/mL lysozyme solution (VWR) and 2.1 μL (10.5 U) lysostaphin (Sigma-Aldrich) for 30 min, and DNA was eluted in 100 μL of nuclease-free water. Each DNA extraction included a negative control without bacteria. DNA was further purified using the ProNex size-selective purification system with a bead ratio of 1.3× (350-bp cutoff), and DNA was eluted in nuclease-free water.

The ratios of *A*_260_/*A*_280_ and *A*_260_/*A*_230_ were determined on a QIAxpert system (Qiagen) to assess sample purity. DNA concentration was determined on a GloMax plate reader (Promega) using the QuantiFluor One double-stranded DNA (dsDNA) system (Promega). Fragment length distribution was assessed on a QIAxcel Advanced system (Qiagen). Each DNA extract per bacterial isolate and extraction method was split into 3 aliquots and stored at 4°C until shipped to the respective partner laboratories. The boxed DNA samples were shipped on dry ice and arrived within 3 to 6 days to each of the three partner laboratories.

### Initial validation-phase library preparation.

Each partner laboratory prepared libraries for each of the three extraction methods. The rapid barcoding kit (SQK-RBK004) using 400 ng DNA input per sample was used following the manufacturer’s instructions. Each library pool consisted of six isolates per extraction method, and a negative control was run on R10.3 (FLO-MIN111) flow cells (Oxford Nanopore Technologies) on either a MinION or GridION device. R10.3 flow cells were not officially listed to be compatible with the SQK-RBK004 kit; however, compatibility was confirmed by ONT, and the combination was available in the MinKNOW software. R10.3 flow cells were an improvement on R9.4.1 for distinguishing homopolymer regions because of the two reader heads; however, it was at a cost of slightly reducing throughput. Each partner laboratory sequenced 21 samples. Sequencing runs lasted for up to 72 h; basecalling was performed with Guppy 4.3+ using the hac (high accuracy basecalling) mode.

### Initial validation-phase sequencing reads processing, genome assembly, and annotation.

Raw reads of samples with coverage greater than 100× were subsampled to 100× (assuming an average genome size of 6 Mb) using rasusa v0.6.0 (26), quality filtered using NanoFilt (27) with parameter ‘-q 7’, and assembled using Canu v2.1.1 (28). Contigs were polished using Racon (29) for three rounds. Assembled genomes were annotated with Prokka v1.14.5 (30), parameter --mincontiglen 200, and checked for quality and completeness using BUSCO v5.2.2 (31) and QUAST v5.0.2 (32), both with default settings. Filtered reads were mapped to assemblies using BWA-MEM (33), sorted with Picard Tools’s SortSam command (34), converted to BAM format using SAMtools v1.12 (35). Genome coverage was calculated using bedtools genomecov (36).

### Initial validation-phase taxonomic identification and genotyping.

Kraken v2.0.8-beta (37) was run with default settings on the filtered reads using the public minikraken2_v2_201904 database. The species with the highest proportion of assigned reads was picked as taxonomic assignment. Core genome multilocus sequence typing (cgMLST) was run using chewBBACA v2.0.5 (38) against schemas from https://cgmlst.org/ncs. PlasmidFinder was used for plasmid (replicon) typing (39).

### Initial validation-phase antimicrobial resistance marker detection.

Alignment-based detection of AMR resistance markers was performed by a six-frame translation of an assembled genome, comparing all translated open reading frames against the ARESdb AMR marker reference database using Diamond (40) with a minimum query coverage of 60% and a minimum identity of 90%.

### Initial validation-phase predictive WGS antimicrobial susceptibility testing.

Predictive antimicrobial susceptibility testing from genome assemblies (WGS-AST) was determined by machine learning models as described previously (41, 42). In brief, classification models were trained per species-antimicrobial pair on the AMR reference database ARESdb (24) and supplemented with rule-based classifiers to predict the susceptible or resistant AST interpretive category. The intermediate category was treated as resistant. The database contained genotype (WGS) to phenotype (AST) associations for all six species. The model consisted of extreme gradient boosting, elastic net-regularized logistic regression, and set-covering machine models. Models trained on unbalanced data sets (Shannon index < 0.4), few isolates (*n* < 50), or with high technical uncertainty (low confidence) were discarded after training. Susceptible or resistant was defined following CLSI document M100 ED29 (43).

### Initial validation-phase analysis.

Definitions for accuracy and reproducibility, as described by Kozyreva et al. (44), were adapted for pathogen identification, cgMLST, plasmid replicon detection, and AMR marker detection. The analytical performance of applied assays (taxonomic identification, AMR marker detection, and WGS-AST) was assessed by downsampling of all validation samples. The required base pair counts for sequencing depths of 40×, 30×, 20×, and 10× ONT sequencing were determined based on the size of the reference genome for each species. The LOD was defined as the coverage at which the assay results began to differ from the public reference genomes. The three extraction methods were compared across partner labs, assessing per-base and assembly quality scores, sequencing yield, taxonomic ID, plasmid replicon detection, cgMLST, AMR detection, and predictive WGS-AST.

### Subsequent application phase.

In the application phase, 42 clinical isolates consisting of the same 6 bacterial species recovered from blood cultures from July to August 2021 were used for the workflow validation (Fig. 1). Isolates were sequentially collected at The Johns Hopkins Hospital (Baltimore, MD) and processed according to the validated workflow established in the initial phase. The use of deidentified isolates was exempt from the institutional review board (IRB). Sample collection of individual species was restricted to a maximum of 5 Gram-positive organisms and up to 10 Gram-negative organisms to avoid overrepresentation during sequential sample collection. Gram-negative organisms are arguably more challenging for analysis and were thus given greater weights as follows: A. baumannii (2 samples), E. faecium (5 samples), E. coli (10 samples), K. pneumoniae (10 samples), S. aureus (5 samples), and P. aeruginosa (10 samples). Isolates were stored at −80°C in Microbank tubes (Pro-Lab Diagnostics, Richmond Hill, ON, Canada) until further testing. Bacterial cultivation, reference organism identification and antimicrobial susceptibility testing, DNA extraction, library preparation, and sequencing were carried out at Johns Hopkins University (JHU). Data were assessed for taxonomic identification and WGS-AST compared to culture-based results as defined below.

### Subsequent application-phase organism identification and AST.

Isolates were subcultured from frozen stocks twice on tryptic soy agar with 5% sheep blood (BD Diagnostics, Sparks, MD) for 18 to 24 h at 37°C. The same subculture was used for species identification, broth microdilution antimicrobial susceptibility testing (BMD-AST), and WGS analysis. Bacterial genus and species were identified using matrix-assisted laser desorption ionization–time of flight mass spectrometry (MALDI-TOF MS; Bruker Daltonics Inc., Billerica, MA). BMD-AST results were generated by the lyophilized Sensititre BMD panels GN7F for Gram-negative bacteria and GPN3F for Gram-positive bacteria (Thermo Fisher Scientific, Waltham, MA). BMD-AST data were performed in triplicates, and results were interpreted according to CLSI performance standards (45). BMD-AST replicate measurements that resulted in discordant interpretations were discarded. Isolates with an intermediate AST result or which are intrinsically resistant were treated as resistant. For all BMD-AST studies, quality control organisms were evaluated each day of testing.

### Subsequent application-phase DNA extraction, library preparation, and sequencing.

WGS methods were performed as described above. All extractions were performed with the DNeasy PowerSoil Pro kit (Qiagen) based on the initial validation phase.

### Subsequent application-phase analysis.

The FASTQ files generated at JHU were shared with Ares Genetics, who completed the analysis. The validated workflow was implemented in AREScloud (24) for automated end-to-end processing of ONT sequencing data. WGS-AST results were downloaded from the web application and compared to BMD-AST results to determine categorical agreement (CA). Susceptible and resistance predictions (S/R) were classified either as true positive (TP) (i.e., both WGS-AST and BMD-AST indicated resistance), false positive (FP) (i.e., WGS-AST indicated resistance, whereas BMD-AST indicated susceptibility), true negative (TN) (i.e., both WGS-AST and BMD-AST indicated susceptibility), or false negative (FN) (i.e., WGS-AST indicated susceptibility, whereas BMD-AST indicated resistance). The very major error (VME), major error (ME), and minor error (mE) were defined following CLSI document M52 ED1 (43). BMD-AST results within the intermediate interpretive category always resulted in minor errors because the WGS-AST models only predicted susceptibility and resistance.

### Data availability.

The data set has been deposited at the NCBI under BioProject accession number PRJNA809755. Raw sequence data for each validation sample were deposited at the Sequence Read Archive (SRA) under accession numbers SRR18117831 to SRR18117880.
