# Supplementary material for: Validation and Application of Long-Read Whole-Genome Sequencing for Antimicrobial Resistance Gene Detection and Antimicrobial Susceptibility Testing
Source: Antimicrob Agents Chemother. 2022 Dec 19;67(1):e01072-22. doi: 10.1128/aac.01072-22 (PMC9872642; doi:10.1128/aac.01072-22)
Supplement: Supplemental file 1 — Supplemental material. Download aac.01072-22-s0001.pdf, PDF file, 0.6 MB [file aac.01072-22-s0001.pdf]

Sample ID	Reference Species	Reference ID	Database (Accession)
**ID244**	*Acinetobacter baumannii*	ATCC BAA-1605	ATCC Genome Portal (n.a.)
**ID245**	*Pseudomonas aeruginosa*	ATCC 27853	ATCC Genome Portal (n.a.)
**ID246**	*Klebsiella quasipneumoniae*	ATCC 700603	ATCC Genome Portal (n.a.)
**ID247**	*Staphylococcus aureus*	ATCC BAA-2312	ATCC Genome Portal (n.a.)
**ID248**	*Escherichia coli*	ATCC 35218	ATCC Genome Portal (n.a.)
**ID249**	*Enterococcus faecium*	ATCC 700221	RefSeq (GCF_001594345.1)

Supplementary Table 1: Reference species for the workflow validation.

Partner Lab	Extraction	Reads	Bases	N50	PHRED	Median Length
A	PowerSoil	2115034	7768310140	6087	11.43	2524
B	PowerSoil	2548646	8881712547	5843	11.05	2319
C	PowerSoil	1193160	8012554326	5495	11.97	2319
A	MagAttract	1578984	8334985587	10689	11.15	2949
B	MagAttract	741671	4549672945*	12110	10.20	3571
C	MagAttract	1224862	8086766586	12803	11.92	3933
A	QIAsymphony	905367	5222377454*	10605	11.36	3487
B	QIAsymphony	1599503	8685278690	10329	11.98	3149
C	QIAsymphony	1397733	8689445038	11604	11.83	3769

*Supplementary Table 2: Workflow validation sequencing summary. * indicates that the sequencing run stopped after ~50 hours.*

Sample ID	Partner Lab	Extraction	Assembly Size	N50	Completeness	# CDS
**ID244**	A	QIAsymphony	4336671	4074990	95.2	4366
**ID245**	A	QIAsymphony	7033802	6870307	97.3	6739
**ID246**	A	QIAsymphony	5631411	5318847	91.2	5657
**ID247**	A	QIAsymphony	2996958	2827554	89.8	3104
**ID248**	A	QIAsymphony	5219701	5149124	95.3	5081
**ID249**	A	QIAsymphony	4036357	2874039	92.6	4631
**ID244**	A	MagAttract	4250550	4082780	96.6	4282
**ID246**	A	MagAttract	5607405	5318825	94.6	5595
**ID247**	A	MagAttract	2891853	2861858	84.5	3176
**ID248**	A	MagAttract	5241329	5140670	97.3	5054
**ID249**	A	MagAttract	4090691	2849618	94.6	4589
**ID244**	A	PowerSoil	4184675	3518534	97.3	4123
**ID245**	A	PowerSoil	6862561	6862561	96.6	6480
**ID246**	A	PowerSoil	5520018	2841141	95.3	5450
**ID247**	A	PowerSoil	2856016	2838130	99.3	2721
**ID248**	A	PowerSoil	5203661	5134265	97.3	5053
**ID249**	A	PowerSoil	3334771	1952872	94.6	3663
**ID244**	B	QIAsymphony	4307815	3526721	99.3	4267
**ID245**	B	QIAsymphony	6868775	6868775	96.6	6478
**ID246**	B	QIAsymphony	5605338	5305777	93.2	5549
**ID247**	B	QIAsymphony	2930330	2849933	91.9	2852
**ID248**	B	QIAsymphony	5210706	5140572	93.2	5092
**ID249**	B	QIAsymphony	3719926	2877697	86.5	4571
**ID244**	B	MagAttract	4322878	4068121	98.0	4313
**ID245**	B	MagAttract	9499381	6869007	97.3	9438
**ID246**	B	MagAttract	5787908	5333045	94.6	5741
**ID247**	B	MagAttract	2962891	2875634	79.1	3340
**ID248**	B	MagAttract	5305990	5148158	90.5	5463
**ID249**	B	MagAttract	3953782	2892526	85.9	4674
**ID244**	B	PowerSoil	4397579	3382470	90.5	4558
**ID245**	B	PowerSoil	7963606	6867049	98.0	7734
**ID246**	B	PowerSoil	5583172	5295174	93.9	5657

**ID247**	B	PowerSoil	3064727	1641089	92.6	3241
**ID248**	B	PowerSoil	5218941	5128149	91.9	5449
**ID249**	B	PowerSoil	4015261	2265820	89.9	4778
**ID244**	C	QIAsymphony	4202229	4116867	96.6	4286
**ID245**	C	QIAsymphony	6858648	6858648	95.3	6493
**ID246**	C	QIAsymphony	5648092	5312632	91.9	5706
**ID247**	C	QIAsymphony	3048632	2864205	91.9	3190
**ID248**	C	QIAsymphony	5220806	5144773	89.2	5135
**ID249**	C	QIAsymphony	3797034	2873010	87.2	4352
**ID244**	C	MagAttract	4201529	1363025	81.8	5230
**ID245**	C	MagAttract	5325761	128768	31.1	7462
**ID246**	C	MagAttract	5690256	5316177	89.9	5955
**ID247**	C	MagAttract	2914669	2842503	91.9	2888
**ID248**	C	MagAttract	5233707	5163256	89.2	5298
**ID249**	C	MagAttract	3704769	2933678	73.7	4844
**ID244**	C	PowerSoil	4257442	4062912	91.2	4486
**ID245**	C	PowerSoil	6870196	6851667	95.3	6659
**ID246**	C	PowerSoil	5558981	5295448	88.5	5680
**ID247**	C	PowerSoil	3057144	2830872	89.2	3229
**ID248**	C	PowerSoil	5525479	5130950	92.6	5789
**ID249**	C	PowerSoil	3517105	2267320	85.8	4243

Supplementary Table 3: Workflow validation assembly summary per sample.

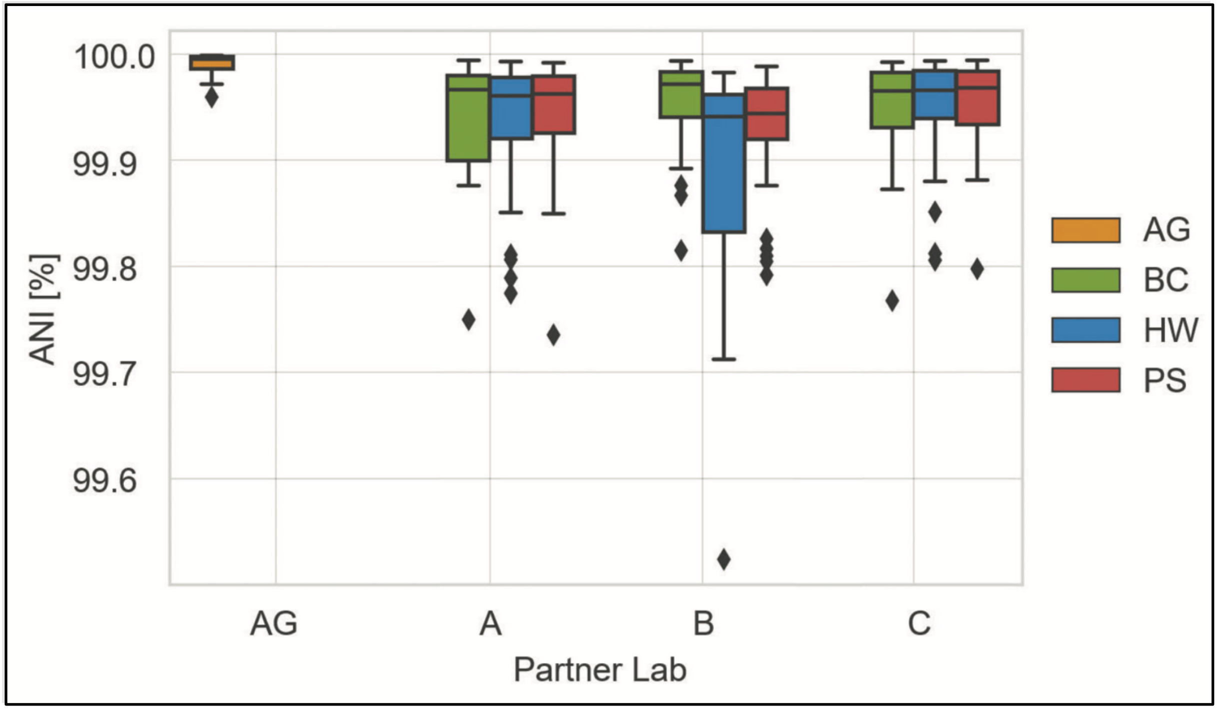

Supplementary Figure 1: Average nucleotide identity (ANI) of the ONT-derived assemblies generated in the validation phase and Illumina assemblies generated in a previous study compared to reference assemblies. AG: Reference assemblies from previous study, PS: DNeasy PowerSoil Pro extraction Kit, HW: MagAttract HMW DNA extraction Kit, BC: QIAsymphony DSP DNA Mini extraction Kit.

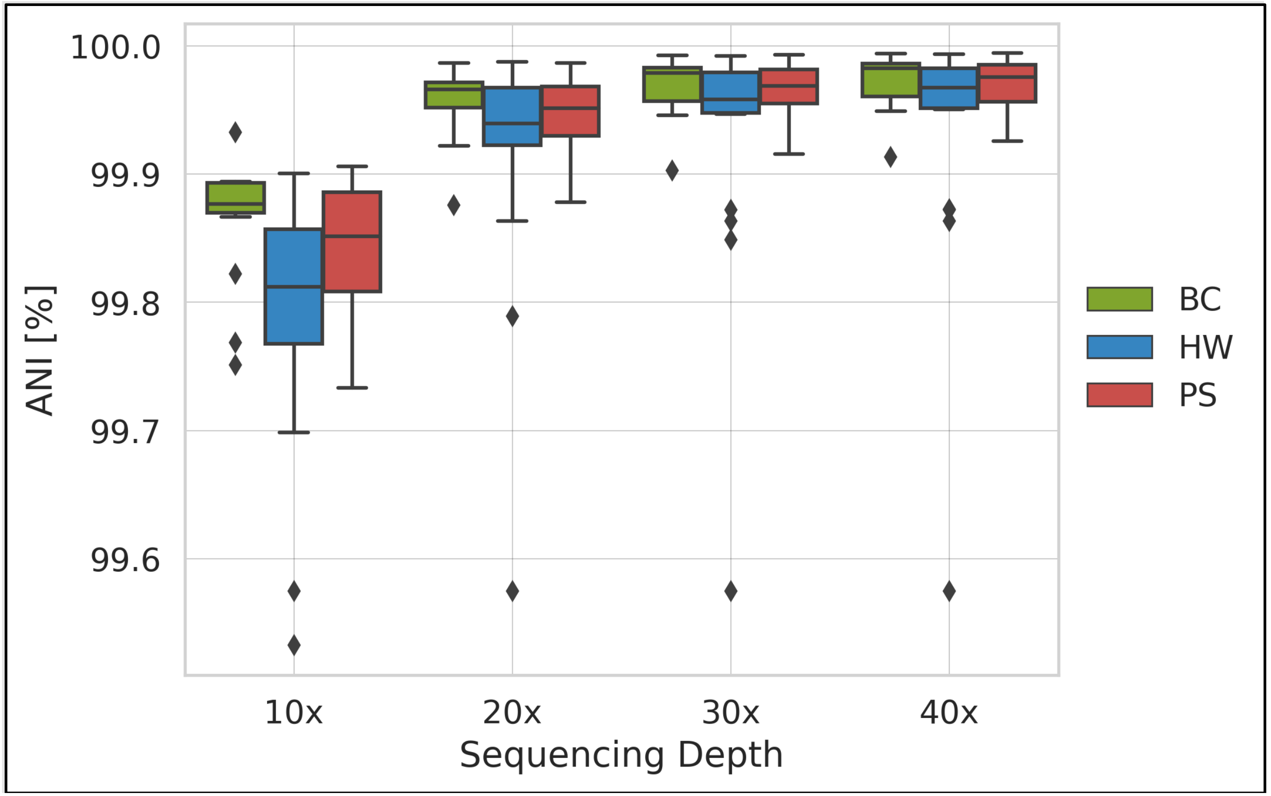

Supplementary Figure 2: Average nucleotide identity (ANI) of the ONT-derived assemblies generated in the validation phase by sequencing depth compared to reference assemblies. PS: DNeasy PowerSoil Pro extraction Kit, HW: MagAttract HMW DNA extraction Kit, BC: QIAsymphony DSP DNA Mini extraction Kit.

	Metric	ID244 (n:5) [%]			ID245 (n:5) [%]			ID246 (n:12) [%]			ID247 (n:8) [%]			ID248 (n:15) [%]			ID249 (n:5) [%]		
		PS	HW	BC	PS	HW	BC	PS	HW	BC	PS	HW	BC	PS	HW	BC	PS	HW	BC
AG	CA	100.0	-	-	100.0	-	-	60.0	-	-	87.5	-	-	100.0	-	-	100.0	-	-
	VME	0.0	-	-	0.0	-	-	33.3	-	-	100.0	-	-	0.0	-	-	0.0	-	-
	ME	0.0	-	-	0.0	-	-	0.0	-	-	0.0	-	-	0.0	-	-	0.0	-	-
	mE	0.0	-	-	0.0	-	-	30.0	-	-	0.0	-	-	0.0	-	-	0.0	-	-
A	CA	100.0	100.0	100.0	75.0	75.0	100.0	60.0	60.0	60.0	87.5	87.5	87.5	100.0	100.0	92.3	100.0	100.0	100.0
	VME	0.0	0.0	0.0	0.0	0.0	0.0	33.3	33.3	33.3	100.0	100.0	100.0	0.0	0.0	0.0	0.0	0.0	0.0
	ME	0.0	0.0	0.0	25.0	25.0	0.0	0.0	0.0	0.0	0.0	0.0	0.0	0.0	0.0	8.3	0.0	0.0	0.0
	mE	0.0	0.0	0.0	0.0	0.0	0.0	30.0	30.0	30.0	0.0	0.0	0.0	0.0	0.0	0.0	0.0	0.0	0.0
B	CA	100.0	100.0	100.0	100.0	25.0	100.0	60.0	60.0	60.0	87.5	87.5	87.5	76.9	100.0	100.0	100.0	100.0	100.0
	VME	0.0	0.0	0.0	0.0	0.0	0.0	33.3	33.3	33.3	100.0	100.0	100.0	0.0	0.0	0.0	0.0	0.0	0.0
	ME	0.0	0.0	0.0	0.0	75.0	0.0	0.0	0.0	0.0	0.0	0.0	0.0	25.0	0.0	0.0	0.0	0.0	0.0
	mE	0.0	0.0	0.0	0.0	0.0	0.0	30.0	30.0	30.0	0.0	0.0	0.0	0.0	0.0	0.0	0.0	0.0	0.0
C	CA	100.0	100.0	100.0	100.0	75.0	100.0	60.0	60.0	60.0	87.5	87.5	87.5	100.0	100.0	100.0	100.0	100.0	100.0
	VME	0.0	0.0	0.0	0.0	0.0	0.0	33.3	33.3	33.3	100.0	100.0	100.0	0.0	0.0	0.0	0.0	0.0	0.0
	ME	0.0	0.0	0.0	0.0	25.0	0.0	0.0	0.0	0.0	0.0	0.0	0.0	0.0	0.0	0.0	0.0	0.0	0.0
	mE	0.0	0.0	0.0	0.0	0.0	0.0	30.0	30.0	30.0	0.0	0.0	0.0	0.0	0.0	0.0	0.0	0.0	0.0

Supplementary Table 4: Performance of WGS-AST for workflow validation. AG: Ares Genetics Illumina validation study dataset, A: partner lab A, B: partner lab B, C: partner lab C; CA: categorical agreement, VME: very major error, ME: major error, mE: minor error. PS: DNeasy PowerSoil Pro extraction Kit, HW: MagAttract HMW DNA extraction Kit, BC: QIAsymphony DSP DNA Mini extraction Kit; The total number of compounds tested is given in brackets in the column header.

Sample ID	Reads	Bases	N50	PHRED	Median Length
**Efae-Saur_bc01**	49408	194303960	6461	11.73	2620
**Efae-Saur_bc02**	83561	358865946	7577	11.62	2720
**Efae-Saur_bc03**	51866	212168152	7300	11.53	2563
**Efae-Saur_bc04**	67967	288269361	7348	11.58	2778
**Efae-Saur_bc05**	116493	442297942	6243	11.64	2529
**Efae-Saur_bc06**	81500	567494972	12358	11.93	4337.5
**Efae-Saur_bc07**	69058	468140762	11864	12.11	4318
**Efae-Saur_bc08**	68242	393559048	10135	11.92	3571.5
**Efae-Saur_bc09**	56092	413357589	13134	11.88	4624
**Efae-Saur_bc10**	48073	301386818	11052	11.97	3933
**Eclo-Abau_bc09**	114327	683755314	10659	12.41	3661
**Eclo-Abau_bc10**	68235	392104078	10515	12.50	3464
**Ecol_bc01**	62102	335128664	10263	10.89	3161
**Ecol_bc02**	94382	567197509	11104	10.82	3635
**Ecol_bc03**	61695	393559034	11842	10.85	3819
**Ecol_bc04**	75208	496538247	12236	10.86	3985.5
**Ecol_bc05**	101074	698037931	13368	10.89	3923
**Ecol_bc06**	110935	627606534	10195	10.78	3502
**Ecol_bc07**	64451	417123781	11967	10.96	3966
**Ecol_bc08**	81843	498792381	11652	10.89	3565
**Ecol_bc09**	65918	483279391	13495	10.90	4499.5
**Ecol_bc10**	65252	395723498	11751	10.74	3438
**Kpne_bc01**	139486	758448365	10667	10.72	3137
**Kpne_bc02**	154247	854230528	10733	10.69	3319
**Kpne_bc03**	139588	872960712	12791	10.66	3487
**Kpne_bc04**	130049	937790586	14448	10.60	4059
**Kpne_bc05**	186143	1147208243	11902	10.79	3609
**Kpne_bc06**	159578	1087065569	12949	10.75	4072
**Kpne_bc07**	156553	1031064749	13752	10.66	3675
**Kpne_bc08**	162233	901869333	10629	10.74	3264
**Kpne_bc09**	118077	760508900	12407	10.80	3830

**Kpne_bc10**	115061	659483409	11109	10.84	3396
**Paer_bc01**	46615	278286032	11745	12.38	3341
**Paer_bc02**	77363	436769929	10702	12.36	3351
**Paer_bc03**	74559	439919101	11060	12.20	3549
**Paer_bc04**	91537	465691053	9548	12.23	3092
**Paer_bc05**	137170	834244317	12560	12.30	3172
**Paer_bc06**	94585	607973569	12883	12.40	3551
**Paer_bc07**	125172	691139721	9832	12.27	3489
**Paer_bc08**	93528	466809876	11109	12.09	2355
**Paer_bc09**	48803	371225353	14701	12.44	4391
**Paer_bc10**	75461	473127017	12307	12.40	3477

Supplementary Table 5: Application set sequencing summary per sample.

Sample ID	Assembly	N50	Completeness	# CDS
**Eclo-Abau_bc09**	4146506	3973587	98	4048
**Eclo-Abau_bc10**	3762427	3710607	98.6	3568
**Ecol_bc01**	5540761	5255736	93.9	5965
**Ecol_bc02**	5133300	5123958	93.2	5182
**Ecol_bc03**	5415225	5183046	93.3	5569
**Ecol_bc04**	5271739	5063900	97.3	5421
**Ecol_bc05**	5489219	5285282	94.6	5758
**Ecol_bc06**	5499957	5187930	91.2	5863
**Ecol_bc07**	5460120	5031195	93.2	5650
**Ecol_bc08**	4757863	2625913	96.6	4826
**Ecol_bc09**	5558297	4771151	93.9	5938
**Ecol_bc10**	5116440	4836817	94.6	5364
**Efae-Saur_bc01**	3455123	3002076	98.7	3535
**Efae-Saur_bc02**	2891279	2637850	98	2940
**Efae-Saur_bc03**	2678833	2461767	96.6	2712
**Efae-Saur_bc04**	3262352	2833356	97.3	3346
**Efae-Saur_bc05**	3655039	2950593	99.3	3832
**Efae-Saur_bc06**	3002738	2945636	98	2885
**Efae-Saur_bc07**	2918627	2911951	99.3	2763
**Efae-Saur_bc08**	2880054	2790857	100	2722
**Efae-Saur_bc09**	3142322	1639771	98.6	3106
**Efae-Saur_bc10**	2753846	2753846	98	2568
**Kpne_bc01**	5842976	5207443	84.5	6642
**Kpne_bc02**	5840276	1888750	82.5	6624
**Kpne_bc03**	5837222	5492990	83.1	6827
**Kpne_bc04**	5688890	4580845	80.4	6485
**Kpne_bc05**	5582575	5342765	82.4	6186
**Kpne_bc06**	5863777	3068770	86.5	6569
**Kpne_bc07**	5512874	5139987	83.8	6150
**Kpne_bc08**	5467264	5236936	84.5	6094
**Kpne_bc09**	6367580	1670064	84.5	7449
**Kpne_bc10**	5516923	5313286	85.1	6142

**Paer_bc01**	6310245	6310245	91.2	6157
**Paer_bc02**	6662736	6662736	95.3	6520
**Paer_bc03**	7028619	6952116	94.6	6742
**Paer_bc04**	6438167	6438167	96.7	6126
**Paer_bc05**	6978974	6978974	96	6698
**Paer_bc06**	6569828	6569828	97.3	6241
**Paer_bc07**	6975098	5669395	92.6	6797
**Paer_bc08**	6724869	6591926	95.9	6522
**Paer_bc09**	7314609	7266612	93.9	7091
**Paer_bc10**	6437685	6437685	97.9	6076

Supplementary Table 6: Application set assembly summary per sample.

Isolate ID	Pathogen	β-lactamase genes	Other (including proteins with variants)
**Eclo-Abau_bc09**	*A. baumannii*	beta-lactamase, OXA-66, OXA-23	aarA, AbaF, AbaQ, AbeM, AbeS, AcrB, AdeA, AdeB, AdeC, AdeF, AdeG, AdeH, AdeI, AdeJ, AdeK, AdeL, AdeN, AdeR, AdeS, AmvA, ANT(3'')-IIa, APH(3'')-Ib, APH(6)-Id, ArmA, BaeR, BaeS, CAT, DNA_topoisomerase_subunit, EmrB, iucC, iutA, MacA, MacB, MerR, Mph(D), MsbA, MsrE, mymA, NreB, OprM, outC, parC, RlmN, RND_efflux_transporter, smvA, sodC, SoxR, Sul2, Tet(A), tipA, TolC, nemR
**Eclo-Abau_bc10**	*A. baumannii*	ADC-158, OXA-180	aarA, AbaF, AbaQ, AbeM, AbeS, AcrB, AdeA, AdeB, AdeF, AdeG, AdeH, AdeI, AdeJ, AdeK, AdeL, AdeN, AdeR, AdeS, AmvA, ANT(3'')-IIa, BaeR, BaeS, CAT, EmrB, iucC, iutA, MacA, MacB, MerR, MsbA, mymA, NreB, OprM, parC, RlmN, RND_efflux_transporter, smvA, sodC, SoxR, Tet(A), tipA, TolC, nemR
**Ecol_bc01**	*E. coli*	ampH, CTX-M-15, EC-145, OXA-1, TEM-1	AAC(3)-IIc, AAC(6')-Ib-cr, aadA5, AcrA, AcrB, AcrD, AcrE, AcrS, ampR, APH(3'')-Ib, APH(6)-Id, argR, BacA, BaeR, BaeS, CatB3, CNF1, CpxA, CRP, cusF, cusR, cycA, Dfr, eae, EmrA, EmrB, EmrD, EmrE, EmrK, EmrY, EvgA, EvgS, fdeC, fepA, fliA, fliG, fliI, fliQ, fliY, fliZ, GadW, GadX, gyrA, gyrA, H-NS, IucA, KdpE, MacA, MarA, MarR, MarR, MdfA, mdlB, MdtB, MdtC, MdtE, MdtG, MdtH, MdtM, MdtN, MdtO, MdtP, MgrB, Mph(A), Mrx, MsbA, MsrB, OmpC, ompR, papF, PapG-III, PapH, parC, parC, parC, parE, PatA, PhoQ, PmrC, PmrE, PmrF, PmrL, PtsI, RosB, rpoS, Rpsl, sfaH, SoxR, SoxR, srpC, Sul1, Sul2, Tet(A), TmrB, TolC, UhpT, YbtP, YbtQ, yheI, YojI, qacEdelta1
**Ecol_bc02**	*E. coli*	EC-145, ampC1	AcrA, AcrB, AcrD, AcrE, AcrF, AcrS, argR, BacA, BaeR, BaeS, CpxA, CRP, cusF, cusR, CyaA, cycA, eae, EmrA, EmrB, EmrD, EmrE, EmrK, EmrY, EspX1, EvgA, EvgS, fdeC, fepA, fimD, flhA, fliA, fliG, fliI, fliQ, fliY, fliZ, GadW, GadX, H-NS, inlJ, IucA, iutA, KdpE, LpfA, MacA, MarA, MarR, MarR, MdfA, mdlB, MdtA, MdtB, MdtC, MdtE, MdtF, MdtG, MdtH, MdtM, MdtN, MdtO, MdtP, MgrB, Mph(B), MsbA, MsrB, OmpC, ompR, PapC, papF, PapG-III, PapH, parC, PatA, PhoQ, PmrC, PmrE, PmrF, PmrL, RosB, rpoS, Rpsl, sfaH, SoxR, SoxR, SoxR, TolC, YbtP, YbtQ, YojI, yheI
**Ecol_bc03**	*E. coli*	ampH, EC-145, TEM-1	AcrA, AcrB, AcrD, AcrE, AcrS, ampR, argR, BacA, BaeR, BaeS, CRP, cusF, cusR, EmrA, EmrB, EmrD, EmrE, EmrK, EmrY, EvgA, EvgS, fdeC, fepA, flhA, fliA, fliG, fliI, fliQ, fliZ, GadW, GadX, gspA, H-NS, IroE, IroN, IucA, KdpE, MacA, MarA, MarR, MarR, MarR, MdfA, mdlB, MdtA, MdtB, MdtC, MdtE, MdtF, MdtG, MdtH, MdtM, MdtN, MdtO, MdtP, MgrB, MsbA, MsrB, OmpC, ompR, PapC, PapE, parC, PatA, PhoQ, PmrC, PmrE, PmrF, PmrL, RosB, rpoS, Rpsl, sfaH, SoxR, SoxR, TolC, YbtP, YbtQ, YojI, yheI
**Ecol_bc04**	*E. coli*	ampH, EC-145, TEM-1	AcrA, AcrB, AcrD, AcrE, AcrS, ampR, argR, BacA, BaeR, BaeS, CpxA, CRP, cusF, cusR, EmrA, EmrB, EmrD, EmrE, EmrK, EmrY, EvgA, EvgS, fdeC, fepA, flhA, fliA, fliG, fliI, fliQ, fliY, fliZ, GadW, GadX, GlpT, GlpT, gspA, H-NS, KdpE, MacA, MacB, MarA, MarR, MarR, MarR,

			MdfA, mdlB, MdtA, MdtB, MdtC, MdtE, MdtF, MdtG, MdtH, MdtN, MdtO, MdtP, MgrB, MsbA, MsrB, OmpC, ompR, PapC, PapE, papF, PapG-III, PapH, parC, PatA, PhoQ, PmrC, PmrE, PmrL, RosB, rpoS, Rpsl, SenB, sfaH, SoxR, SoxR, TolC, YbtP, YbtQ, YojI, yheI
**Ecol_bc05**	*E. coli*	ampC1, ampH, EC-145, TEM-84, TEM-1	AcrA, AcrB, AcrD, AcrE, AcrF, AcrS, ampR, APH(3'')-Ib, APH(6)-Id, argR, BacA, BaeR, BaeS, CpxA, CRP, cusF, cusR, CyaA, eae, EmrA, EmrB, EmrD, EmrK, EmrY, EvgA, EvgS, fdeC, fepA, fimD, flhA, fliA, fliG, fliI, fliQ, fliY, fliZ, GadW, GadX, GlpT, GlpT, GlpT, GlpT, GlpT, gspA, H-NS, inlJ, KdpE, LpfA, MacA, MacB, MarA, MarR, MarR, MdfA, mdlB, MdtA, MdtB, MdtC, MdtE, MdtF, MdtG, MdtH, MdtN, MdtO, MgrB, Mph(B), MsbA, MsrB, OmpC, ompR, PapC, papF, PapH, parC, PatA, PhoQ, PmrC, PmrE, PmrF, PmrL, RosB, rpoS, Rpsl, SenB, sfaH, SoxR, SoxR, SoxR, TolC, UhpT, YbtP, YbtQ, yheI, YojI, Sul2
**Ecol_bc06**	*E. coli*	ampH, TEM-1	aadA5, AcrA, AcrB, AcrE, AcrS, ampR, APH(3'')-Ib, APH(6)-Id, argR, BacA, BaeR, BaeS, CNF1, CpxA, CRP, cusF, cusR, Dfr, eae, EmrA, EmrB, EmrD, EmrE, EmrK, EmrY, EvgA, fdeC, fepA, flhA, fliA, fliG, fliI, fliQ, fliY, fliZ, GadW, GadX, gyrA, H-NS, IucA, iutA, KdpE, MacA, MacB, MarA, MarR, MarR, mdlB, MdtA, MdtB, MdtC, MdtE, MdtF, MdtG, MdtH, MdtM, MdtN, MdtO, MdtP, MgrB, Mph(A), MsbA, MsrB, NfaE, OmpC, ompR, papF, PapH, parC, parE, PatA, PhoQ, PmrC, PmrE, PmrF, PmrL, PtsI, RosB, rpoS, Rpsl, SenB, sfaH, SoxR, SoxR, srpC, Sul1, Sul2, Tet(A), TolC, UhpT, YbtP, YbtQ, yheI, YojI, qacEdelta1
**Ecol_bc07**	*E. coli*	ampH, EC-145, TEM-1	AcrA, AcrB, AcrD, AcrE, AcrS, ampR, argR, BacA, BaeR, BaeS, CNF1, CpxA, CRP, cusF, cusR, EmrA, EmrB, EmrD, EmrE, EmrK, EmrY, EvgA, fdeC, fepA, flhA, fliA, fliG, fliI, fliQ, fliY, fliZ, FocG, GadW, GadX, galF, gspA, H-NS, IbeA, IroE, IroN, KdpE, MacA, MacB, MarA, MarR, MarR, MdfA, mdlB, MdtA, MdtB, MdtC, MdtE, MdtF, MdtG, MdtH, MdtN, MdtO, MdtP, MgrB, MsbA, MsrB, OmpC, ompR, parC, PatA, PhoQ, PmrC, PmrF, PmrL, RosB, rpoS, Rpsl, SenB, SfaF, sfaH, SoxR, SoxR, TolC, vactox, YbtP, YbtQ, YojI, yheI
**Ecol_bc08**	*E. coli*	ampH, EC-145, ampC1	AcrA, AcrB, AcrD, AcrE, AcrF, AcrS, ampR, argR, BacA, BaeR, BaeS, CpxA, CRP, cusF, cusR, cycA, eae, EmrA, EmrB, EmrD, EmrK, EmrY, EspX1, EvgA, fepA, fimD, flhA, fliA, fliG, fliI, fliQ, fliY, fliZ, GadW, GadX, H-NS, inlJ, KdpE, MacA, MarA, MdfA, mdlB, MdtA, MdtB, MdtC, MdtE, MdtF, MdtG, MdtH, MdtM, MdtN, MdtO, MdtP, MgrB, Mph(B), MsbA, MsrB, OmpC, ompR, parC, PatA, PhoQ, PmrC, PmrF, PmrL, RosB, rpoS, Rpsl, sfaH, SoxR, TolC, YbtP, YbtQ, YojI, yheI
**Ecol_bc09**	*E. coli*	ampH, EC-145, OXA-1, CTX-M-15	AAC(3)-IIc, AAC(6')-Ib-cr, AcrA, AcrB, AcrE, AcrS, ampR, argR, BacA, BaeR, CNF1, CpxA, CRP, cusF, cusR, cycA, EmrA, EmrB, EmrD, EmrE, EmrK, EmrY, EvgA, EvgS, fdeC, fepA, flhA, fliA, fliG, fliI, fliQ, fliY, fliZ, GadW, GadX, gyrA, gyrA, H-NS, IucA, KdpE, MacA, MarA, MarR, MarR, MdfA, mdlB, MdtA, MdtB, MdtC, MdtE, MdtF, MdtG, MdtH, MdtM, MdtN, MdtO, MdtP, MgrB, MsbA, MsrB, OmpC, ompR, PapC, papF, PapG-III, PapH, parC, parC, parC,

			parE, PatA, PhoQ, PmrC, PmrE, PmrF, PmrL, PtsI, RosB, rpoS, Rpsl, sfaH, SoxR, SoxR, Tet(A), TmrB, TolC, UhpT, Vga(C), YbtP, YbtQ, YojI, CatB3
**Ecol_bc10**	*E. coli*	ampH, EC-145, CTX-M-27	AcrA, AcrB, AcrD, AcrE, AcrS, ampR, argR, BacA, BaeR, BaeS, CpxA, CRP, eae, EmrA, EmrB, EmrD, EmrE, EmrK, EmrY, EvgA, EvgS, fdeC, fepA, flhA, fliA, fliG, fliI, fliQ, fliY, fliZ, GadW, GadX, gyrA, gyrA, H-NS, IucA, KdpE, MacA, MarA, MarR, MarR, MdfA, mdlB, MdtA, MdtB, MdtC, MdtE, MdtF, MdtG, MdtH, MdtM, MdtN, MdtO, MdtP, MgrB, MsbA, MsrB, OmpC, ompR, parC, parC, parE, PatA, PhoQ, PmrC, PmrE, PmrF, PmrL, PtsI, RosB, rpoS, Rpsl, sfaH, SoxR, SoxR, Tet(A), TolC, UhpT, YbtP, YbtQ, YojI, yheI
**Efae-Saur_bc01**	*E. faecium*		AAC(6'), AAC(6')-Ie-APH(2'')-Ia, ANT(6)-Ia, APH(3')-IIIa, DfrF, EfmA, ErmB, gyrA, gyrA, parC, parC, PBP5, PBP5, PBP5, PBP5, PBP5, PBP5, PBP5, PBP5, PBP5, PBP5, PBP5, PBP5, PBP5, PBP5, PBP5, PBP5, PBP5, Sat4, Str, VanA, VanHA, VanRA, VanSA, VanXA, VanYA, VanZA, DfrG
**Efae-Saur_bc02**	*E. faecium*		AAC(6'), Eat(A), gyrA, parC, Str, MsrC
**Efae-Saur_bc03**	*E. faecium*		AAC(6'), Eat(A), gyrA, MsrC, parC, PBP5, PBP5, PBP5, PBP5, PBP5, PBP5, PBP5, PBP5, PBP5, PBP5, PBP5, Str, EfmA
**Efae-Saur_bc04**	*E. faecium*		AAC(6'), DfrF, gyrA, gyrA, MsrC, parC, parC, PBP5, PBP5, PBP5, PBP5, PBP5, PBP5, PBP5, PBP5, PBP5, PBP5, PBP5, PBP5, PBP5, PBP5, PBP5, PBP5, Str, Tet(L), tetM, EfmA
**Efae-Saur_bc05**	*E. faecium*		AAC(6'), AAC(6')-Ie-APH(2'')-Ia, ANT(6)-Ia, APH(3')-IIIa, DfrF, EfmA, ErmB, gyrA, gyrA, MsrC, parC, parC, PBP5, PBP5, PBP5, PBP5, PBP5, PBP5, PBP5, PBP5, PBP5, PBP5, PBP5, PBP5, PBP5, PBP5, PBP5, PBP5, PBP5, Sat4, Str, VanA, VanHA, VanRA, VanSA, VanXA, VanYA, VanZA, DfrG
**Efae-Saur_bc06**	*S. aureus*	BlaZ	aad(6), ABC_efflux_pump, APH(3')-IIIa, ArlR, ArlS, BlaI, BlaR1, DfrG, eamA, ebh, EmrB, ErmC, FosB, gyrA, ktrB, MecA, MecR1, MepA, MepR, MgrA, Mph(C), MprF, MsrA, MurA, NorA, norG, parC, RlmN, RND_efflux_transporter, Sat4, SAV1866, sbi, scn, sdrC, Tet(38), SrmB
**Efae-Saur_bc07**	*S. aureus*		ABC_efflux_pump, ArlR, ArlS, eamA, ebh, EmrB, FosB, gyrA, ktrB, MecA, MecR1, MepA, MepR, MgrA, MprF, MurA, NorA, norG, RlmN, RND_efflux_transporter, SAV1866, sbi, scn, sdrC, Tet(38), SrmB
**Efae-Saur_bc08**	*S. aureus*	BlaZ	ABC_efflux_pump, ArlR, ArlS, BlaI, BlaR1, eamA, ebh, EmrB, FosB, ktrB, MecA, MecR1, MepA, MepR, MgrA, Mph(C), MprF, MsrA, MurA, NorA, norG, RlmN, RND_efflux_transporter, SAV1866, sbi, scn, sdrC, Tet(38), SrmB
**Efae-Saur_bc09**	*S. aureus*	BlaZ	aad(6), ABC_efflux_pump, ANT(4')-Ib, ANT(9)-Ia, APH(3')-IIIa, ArlR, ArlS, BlaI, BlaR1, bleO, cycA, eamA, EmrB, entD, ErmA, FosB, gyrA, icaB, IleS, KdpE, ktrB, MecA, MecI, MecR1,

			MepA, MepR, MgrA, Mph(C), MprF, MsrA, NorA, norG, parC, parC, RlmN, RND_efflux_transporter, Sat4, SAV1866, sbi, Tet(38), SrmB
**Efae-Saur_bc10**	*S. aureus*	BlaZ	ABC_efflux_pump, ArlR, ArlS, BlaI, EmrB, ErmT, GlpT, ktrB, MepA, MepR, MgrA, MprF, MurA, MurA, NorA, parE, RlmN, RND_efflux_transporter, SAV1866, scn, sdrC, Tet(38), sbi
**Kpne_bc01**	*K. pneumoniae*	SHV-11	aadA2, AcrA, AcrB, AcrD, AcrR, AcrR, AcrR, AcrR, AcrR, AcrR, AcrR, argR, BaeR, BaeS, bamB, CatA10, CpxA, CRP, cusR, cycA, DfrA12, EmrD, feoB, fimD, fliY, FosA, GlpT, H-NS, KdeA, KpnE, KpnF, KpnG, KpnH, leuO, MarA, MdtC, MerR, Mph(A), MsbA, mtfA, OmpK35, OmpK36, OmpK36, OmpK36, OmpK36, OmpK36, OmpK36, OmpK36, OmpK36, OmpK36, OmpK36, OmpK36, ompR, OqxA, OqxB25, PatA, PmrE, ptxD, RamA, rnfC, RosA, RosB, rpoS, Rpsl, setB, sfaG, SilA, SilB, SilC, srpC, Sul2, tesA, Tet(D), UhpT, Vga(C), virB, qacEdelta1
**Kpne_bc02**	*K. pneumoniae*	SHV-112, LEN-6	AcrA, AcrB, AcrD, argR, BaeR, BaeS, bamB, CpxA, CRP, cusR, cycA, EmrD, fimD, fliY, FosA, GlpT, H-NS, KdeA, KpnE, KpnF, KpnG, KpnH, leuO, MarA, MdtC, MsbA, mtfA, OmpK35, OmpK36, OmpK36, OmpK36, OmpK36, OmpK36, OmpK36, OmpK36, OmpK36, OmpK36, OmpK36, OmpK36, OmpK36, OmpK36, OmpK36, OmpK36, OmpK36, OmpK36, OmpK36, ompR, OqxA, OqxB32, PatA, PmrE, RamA, rnfC, RosA, RosB, rpoS, Rpsl, sfaG, SilA, SilB, SilC, tesA, UhpT, MdtB
**Kpne_bc03**	*K. pneumoniae*	CTX-M-15, SHV-1, TEM-1	AcrA, AcrB, AcrD, APH(3'')-Ib, APH(6)-Id, argR, BaeR, BaeS, bamB, BasS, CpxA, CRP, cusR, cycA, DfrA14, EmrD, feoB, fimD, fliY, FosA, GlpT, H-NS, KdeA, KpnE, KpnF, KpnH, leuO, MarA, MdtC, MsbA, mtfA, OmpK35, OmpK36, OmpK36, OmpK36, OmpK36, OmpK36, OmpK36, OmpK36, OmpK36, OmpK36, OmpK36, OmpK36, OmpK37, OmpK37, OmpK37, OmpK37, OmpK37, OmpK37, OmpK37, OmpK37, OmpK37, ompR, OqxA11, OqxB19, PatA, PmrE, QnrB1, RamA, rnfC, RosA, RosB, rpoS, Rpsl, sfaG, SilA, SilB, SilC, tesA, Tet(A), UhpT, Vga(C), virB, YbtP, YbtQ, Sul2
**Kpne_bc04**	*K. pneumoniae*	SHV-26	AcrA, AcrB, AcrD, AcrR, AcrR, AcrR, AcrR, AcrR, AcrR, argR, BaeR, BaeS, bamB, CpxA, CRP, cusR, EmrD, feoB, fimD, fliY, FosA, GlpT, H-NS, KdeA, KpnE, KpnF, KpnG, KpnH, leuO, MarA, MdtC, MsbA, mtfA, OmpK35, OmpK36, OmpK36, OmpK36, OmpK36, OmpK36, OmpK36, OmpK36, OmpK36, OmpK36, OmpK36, OmpK37, OmpK37, OmpK37, OmpK37, OmpK37, OmpK37, OmpK37, OmpK37, OmpK37, ompR, PatA, PmrE, RamA, rnfC, RosA, rpoS, Rpsl, sfaG, tesA, Vga(C), virB, cycA
**Kpne_bc05**	*K. pneumoniae*	KPC-2, SHV-27, TEM-1	AcrA, AcrB, AcrD, AcrR, AcrR, AcrR, AcrR, AcrR, AcrR, AcrR, argR, BaeR, BaeS, bamB, CpxA, CRP, cusR, EmrD, feoB, fliY, FosA, GlpT, gyrA, H-NS, KdeA, KpnE, KpnF, KpnG, KpnH, leuO, MarA, MdtC, MsbA, mtfA, OmpK35, OmpK36, OmpK36, OmpK36, OmpK36, OmpK36,

			OmpK36, OmpK36, OmpK36, ompR, OqxA8, OqxB19, PatA, PmrE, RamA, rnfC, RosA, RosB, rpoS, Rpsl, sfaG, SilA, SilB, SilC, tesA, UhpT, cycA
**Kpne_bc06**	*K. pneumoniae*	SHV-28, SHV-1	AcrA, AcrB, AcrD, argR, BaeR, BaeS, bamB, CpxA, CRP, cusR, EmrD, feoB, fimD, fliY, FosA, GlpT, H-NS, hlyB, KdeA, KpnE, KpnF, KpnG, KpnH, leuO, MarA, MdtB, MdtC, MsbA, mtfA, OmpK35, OmpK36, OmpK36, OmpK36, OmpK36, OmpK36, OmpK36, OmpK36, OmpK36, OmpK36, OmpK36, OmpK36, OmpK36, OmpK36, OmpK37, OmpK37, ompR, OqxA, OqxB17, parC, PatA, PmrE, ptxD, RamA, rnfC, RosA, RosB, rpoS, Rpsl, sfaG, SilB, SilC, tesA, UhpT, cycA
**Kpne_bc07**	*K. pneumoniae*	SHV-11, CTX-M-15	aadA2, AcrA, AcrB, AcrR, AcrR, AcrR, AcrR, AcrR, AcrR, AcrR, APH(3'')-Ib, APH(3')-Ia, APH(6)-Id, argR, BaeR, BaeS, bamB, CatII, CpxA, CRP, cusR, cycA, DfrA12, EmrB, EmrD, feoB, fimD, fliY, FosA, GlpT, H-NS, KdeA, KpnE, KpnF, KpnG, leuO, MarA, MdtB, MdtC, Mph(A), Mrx, MsbA, mtfA, OmpK35, OmpK37, OmpK37, ompR, OqxA, OqxB25, PatA, PmrE, QnrS7, RamA, rnfC, RosA, RosB, rpoS, Rpsl, sfaG, SilA, SilB, SilC, Sul1, tesA, Tet(A), UhpT, Sul2
**Kpne_bc08**	*K. pneumoniae*	CTX-M-15, SHV-11, TEM-1	AAC(3)-IIc, AcrA, APH(3'')-Ib, APH(6)-Id, argR, BaeR, BaeS, bamB, BasS, CpxA, CRP, cusR, cycA, DfrA14, EmrD, fimD, fliY, FosA, GlpT, H-NS, KdeA, KpnE, KpnF, KpnG, leuO, MarA, MdtB, MdtC, MsbA, mtfA, OmpK35, OmpK36, OmpK36, OmpK36, OmpK36, OmpK36, OmpK36, OmpK36, OmpK36, OmpK37, OmpK37, OmpK37, OmpK37, OmpK37, OmpK37, OmpK37, OmpK37, OmpK37, ompR, OqxA, OqxB25, PatA, PmrE, QnrB1, RamA, rnfC, RosA, RosB, rpoS, Rpsl, sfaG, SilB, SilC, tesA, TmrB, UhpT, Sul2
**Kpne_bc09**	*K. pneumoniae*	SHV-1	AcrA, AcrB, AcrD, AcrR, AcrR, AcrR, AcrR, AcrR, AcrR, AcrR, argR, BaeR, BaeS, bamB, BasS, CpxA, CRP, cusR, EmrD, fimD, fliY, FosA, GlpT, H-NS, KdeA, KpnE, KpnF, KpnG, KpnH, leuO, MarA, MdtC, MsbA, mtfA, OmpK35, OmpK36, OmpK36, OmpK36, OmpK36, OmpK36, OmpK36, OmpK36, OmpK36, OmpK36, OmpK36, OmpK36, OmpK37, OmpK37, OmpK37, OmpK37, OmpK37, OmpK37, OmpK37, OmpK37, OmpK37, ompR, OqxA11, OqxB19, PatA, PmrE, ptxD, RamA, rnfC, RosA, RosB, rpoS, Rpsl, sfaG, SilA, SilB, SilC, tesA, Tet(D), UhpT, YbtP, YbtQ, cycA
**Kpne_bc10**	*K. pneumoniae*	SHV-11	AcrA, AcrB, AcrD, AcrR, AcrR, AcrR, AcrR, AcrR, AcrR, AcrR, argR, BaeR, bamB, BasS, CpxA, CRP, cusR, cycA, EmrD, feoB, fimD, fliY, FosA, GlpT, H-NS, KdeA, KpnE, KpnF, KpnG, KpnH, leuO, MarA, MdtC, MsbA, mtfA, OmpK35, OmpK36, OmpK36, OmpK36, OmpK36, OmpK36, OmpK36, OmpK36, OmpK36, OmpK36, OmpK36, OmpK36, OmpK37, OmpK37, ompR, OqxA, parC, PatA, PmrE, RamA, rnfC, RosA, RosB, rpoS, Rpsl, sfaG, tesA, UhpT, MdtB

**Paer_bc01**	*P. aeruginosa*	OXA-494, PDC-1	APH(3')-IIb, ArmR, BasS, Bcr1, CatB7, CpxR, czcA, czcS, EmrE, flhF, FosA, iutA, MexA, MexB, MexC, MexE, MexF, MexG, MexH, MexI, MexJ, MexK, MexL, MexM, MexN, MexP, MexQ, MexV, MexW, MexX, MexY, MsbA, MuxA, MuxB, MuxC, NalC, nfuA, NorM, OpmB, OpmD, OpmE, OpmH, OprJ, OprM, OprN, parC, PmrL, rnfD, rtcB, SoxR, sttH, TriA, TriB, TriC, walR, copB
**Paer_bc02**	*P. aeruginosa*	OXA-488, PDC-38	APH(3')-IIb, ArmR, BasR, BasS, Bcr1, CpxR, EmrE, flhF, FosA, iutA, MexA, MexB, MexC, MexD, MexG, MexH, MexI, MexJ, MexK, MexL, MexN, MexP, MexQ, MexV, MexX, MexY, MsbA, NalC, NalC, nfuA, NorM, OpmD, OpmE, OpmH, OprJ, OprM, parC, PmpM, PmrL, RND_efflux_transporter, rnfD, rtcB, SoxR, sttH, TriA, TriB, TriC, walR, copB
**Paer_bc03**	*P. aeruginosa*	OXA-494, PDC-8	APH(3')-IIb, ArmR, BasS, Bcr1, CatB7, CpxR, CrpP, czcA, czcS, EmrE, flhF, FosA, iutA, MexA, MexB, MexC, MexD, MexE, MexF, MexG, MexH, MexI, MexJ, MexK, MexL, MexM, MexN, MexP, MexQ, MexV, MexW, MexX, MexY, MsbA, MuxA, MuxB, MuxC, NalC, NalC, nfuA, NorM, OpmB, OpmD, OpmE, OpmH, OprJ, OprM, OprN, parC, PmpM, PmrL, rnfD, rtcB, SoxR, sttH, TriA, TriB, TriC, walR, copB
**Paer_bc04**	*P. aeruginosa*	OXA-395, PDC-3	APH(3')-IIb, ArmR, BasS, Bcr1, CatB7, CpxR, CrpP, czcA, czcS, EmrE, flhF, FosA, iutA, MexA, MexB, MexC, MexE, MexF, MexG, MexH, MexI, MexJ, MexK, MexL, MexM, MexN, MexP, MexQ, MexV, MexW, MexX, MexY, MsbA, MuxA, MuxB, MuxC, NalC, NalC, nfuA, NorM, OpmB, OpmD, OpmE, OpmH, OprJ, OprM, OprN, parC, parE, PmpM, PmrL, rnfD, rtcB, SoxR, sttH, TriA, TriB, TriC, walR, copB
**Paer_bc05**	*P. aeruginosa*	OXA-488, PDC-275, GES-7	AAC(6')-Ib-Hangzhou, aadA6, APH(3')-IIb, APH(3')-XV, ArmR, BasR, BasS, Bcr1, CatB7, copB, CpxR, czcA, czcS, EmrE, FloR, FosA, gyrA, iutA, MerR, MexA, MexB, MexC, MexD, MexE, MexF, MexG, MexH, MexI, MexJ, MexK, MexL, MexM, MexN, MexP, MexQ, MexV, MexX, MexY, MsbA, MuxA, MuxB, MuxC, NalC, NalC, nfuA, NorM, OpmB, OpmD, OpmE, OpmH, OprJ, OprM, OprN, parC, PBP3, PmpM, PmrL, RND_efflux_transporter, rnfD, rtcB, SoxR, sttH, Sul1, Tet(G), TriA, TriB, TriC, walR, qacEdelta1
**Paer_bc06**	*P. aeruginosa*	OXA-486, PDC-257	APH(3')-IIb, ArmR, BasS, Bcr1, CatB7, CpxR, CrpP, czcA, czcS, EmrE, flhF, FosA, iutA, MexA, MexB, MexC, MexD, MexE, MexF, MexG, MexH, MexI, MexJ, MexK, MexL, MexM, MexN, MexP, MexQ, MexV, MexW, MexX, MexY, MsbA, MuxA, MuxC, NalC, NalC, nfuA, OpmB, OpmD, OpmE, OpmH, OprJ, OprM, OprN, parC, PmpM, PmrL, rnfD, rtcB, SoxR, sttH, TriA, TriB, TriC, walR, copB
**Paer_bc07**	*P. aeruginosa*	OXA-488, PDC-5	APH(3')-IIb, ArmR, BasR, BasS, Bcr1, CatB7, CpxR, CrpP, czcA, czcS, EmrE, FosA, gyrA, iutA, MexA, MexB, MexC, MexD, MexE, MexF, MexG, MexH, MexI, MexJ, MexK, MexL, MexN, MexP, MexQ, MexV, MexW, MsbA, MuxA, MuxB, MuxC, NalC, NalC, nfuA, NorM, OpmB,

			OpmD, OpmE, OpmH, OprJ, OprM, OprN, parC, PmpM, rnfD, rtcB, SoxR, sttH, TriA, TriB, TriC, walR, MerR
**Paer_bc08**	*P. aeruginosa*	OXA-494, PDC-8	APH(3')-IIb, ArmR, BasR, BasS, Bcr1, CatB7, CpxR, CrpP, czcA, czcS, EmrE, flhF, FosA, iutA, MexA, MexB, MexC, MexD, MexE, MexF, MexG, MexH, MexI, MexJ, MexK, MexL, MexM, MexN, MexP, MexQ, MexV, MexX, MexY, MsbA, MuxA, MuxC, NalC, nfuA, NorM, OpmB, OpmD, OpmE, OpmH, OprJ, OprM, OprN, parC, PmpM, PmrL, RND_efflux_transporter, rnfD, rtcB, SoxR, sttH, TriA, TriB, TriC, walR, copB
**Paer_bc09**	*P. aeruginosa*	PDC-3, OXA-50	APH(3')-IIb, ArmR, BasS, Bcr1, CatB7, copB, CpxR, CrpP, czcA, czcS, EmrE, flhF, FosA, gyrA, iutA, MexA, MexB, MexC, MexD, MexE, MexF, MexG, MexH, MexI, MexJ, MexK, MexL, MexM, MexN, MexP, MexQ, MexV, MexW, MexX, MexY, MsbA, MuxA, MuxB, MuxC, NalC, NalC, nfuA, NorM, OpmB, OpmD, OpmE, OpmH, OprJ, OprM, OprN, parC, parE, PmpM, PmrL, rnfD, rtcB, SoxR, sttH, TriA, TriB, TriC, walR, MerR
**Paer_bc10**	*P. aeruginosa*	OXA-494, PDC-31	APH(3')-IIb, ArmR, BasR, BasS, Bcr1, CatB7, CpxR, CrpP, czcA, czcS, EmrE, flhF, FosA, iutA, MexA, MexB, MexC, MexD, MexE, MexF, MexG, MexH, MexI, MexJ, MexK, MexL, MexM, MexN, MexP, MexQ, MexV, MexX, MexY, MsbA, MuxA, MuxB, NalC, NalC, nfuA, NorM, OpmB, OpmD, OpmE, OpmH, OprJ, OprM, OprN, parC, parE, PmpM, PmrL, RND_efflux_transporter, rnfD, rtcB, SoxR, sttH, TriA, TriB, TriC, walR, copB

Supplementary Table 7: AMR markers identified in the application dataset including variants.

Organism	Antimicrobials
A. baumannii	amikacin, ampicillin and sulbactam, cefepime, ceftazidime, ceftriaxone, ciprofloxacin, gentamicin, levofloxacin, piperacillin and tazobactam, sulfamethoxazole and trimethoprim, tetracycline, tobramycin
E. faecium	ampicillin, ciprofloxacin, erythromycin, linezolid, quinupristin and dalfopristin, tetracycline, vancomycin
E. coli	amikacin, ampicillin, ampicillin and sulbactam, aztreonam, cefazolin, cefepime, ceftazidime, ceftriaxone, ciprofloxacin, ertapenem, gentamicin, levofloxacin, piperacillin and tazobactam, sulfamethoxazole and trimethoprim, tetracycline, tobramycin
K. pneumoniae	amikacin, aztreonam, cefazolin, cefepime, ceftazidime, ceftriaxone, ciprofloxacin, ertapenem, gentamicin, levofloxacin, sulfamethoxazole and trimethoprim, tetracycline, tobramycin
P. aeruginosa	amikacin, ciprofloxacin, gentamicin, levofloxacin, meropenem, tobramycin
S. aureus	benzylpenicillin, ciprofloxacin, erythromycin, gentamicin, levofloxacin, linezolid, oxacillin, quinupristin and dalfopristin, tetracycline, vancomycin

Supplementary Table 8: List of species-antimicrobial pairs for WGS-AST in the application phase.
